# Health service contacts for mental health and substance use on release from prison: a retrospective population-based data linkage study

**DOI:** 10.1136/bmjopen-2025-107586

**Published:** 2026-02-04

**Authors:** Catriona Connell, Richard Kjellgren, Jan Savinc, Nadine Dougall, Amanj Kurdi, Jim Watson, Christine Haddow, Ashley Brown, Tessa Parkes, Kate Hunt

**Affiliations:** 1 University of Stirling, Stirling, UK; 2 Edinburgh Napier University, Edinburgh, UK; 3 Scottish Centre for Administrative Data Research, Edinburgh, UK; 4 University of Strathclyde, Glasgow, UK; 5 University of the West of Scotland, Paisley, UK

**Keywords:** ACCIDENT & EMERGENCY MEDICINE, PSYCHIATRY, Health Services Accessibility, Prisons, PUBLIC HEALTH, Substance misuse

## Abstract

**Abstract:**

**Background:**

Mental health and substance use problems among people released from prison contribute substantially to premature mortality and emergency services demand. Understanding of mental health and substance use-related health service contacts prior to these severe and costly outcomes is limited. We assessed mental health and substance use-related contact with multiple services, comparing rates of contact among people released from prison to a matched general population sample who had not recently been in prison.

**Objectives:**

To compare rates of health service contacts for mental health and substance use between people released from prison and a matched general population sample.

**Design:**

We conducted a retrospective cohort study using linked administrative data with nationwide coverage. The cohort contained all people released from any Scottish prison in 2015 (exposed group), and a random general population sample matched (ratio 1:5) on sex, age, postcode and deprivation indices, who had no imprisonment in the 5 years prior (unexposed group). We linked individual-level administrative healthcare (prescriptions, outpatient, inpatient, emergency/unscheduled care: 2010–2020), prison (admissions, releases: 2010–2020) and deaths records (2015–2020). We estimated adjusted incidence rate ratios (aIRRs) with 95% CIs using fixed-effects Poisson regression with cluster-robust standard errors, controlling for time-in-community, pre-index mental health and substance use-related health service contacts, and comorbidities. We stratified models by mental health (MH), substance use (SU) and dual diagnosis (attributable to both MH and SU).

**Setting:**

Scotland.

**Results:**

We linked records for 8313 people released from prison, and 41 213 matched individuals. Mental health and substance use-related contact rates were significantly higher for people released from prison across all services, and particularly for emergency and unscheduled care. aIRRs for ambulance contacts were MH=7.75 (95% CI 5.76 to 10.42), SU=7.58 (95% CI 5.71 to 10.08), dual diagnosis=8.28 (95% CI 6.50 to 10.55); and accident and emergency department contacts were MH=4.88 (95% CI 3.78 to 6.29) and SU=7.98 (95% CI 5.71 to 11.17). aIRRs for community prescriptions were MH=1.80 (95% CI 1.67 to 1.94), SU=5.95 (95% CI 4.83 to 7.32), dual diagnosis=5.33 (95% CI 3.70 to 7.68); drug and alcohol services were 7.13 (95% CI 6.00 to 8.48); and outpatient attendances were 2.61 (95% CI 2.17 to 3.16). aIRRs for 24-hour unscheduled telephone support were MH=7.63 (95% CI 4.93 to 11.83) and SU=8.29 (95% CI 3.99 to 17.22); and out-of-hours general practice were MH=5.14 (95% CI 3.66 to 7.22), SU=5.89 (95% CI 3.11 to 11.14) and dual diagnosis=8.85 (95% CI 2.94 to 26.63). aIRRs for general/acute hospital admissions and day cases were MH=2.97 (95% CI 1.43 to 6.16), SU=7.85 (95% CI 4.42 to 13.91), dual diagnosis=13.11 (95% CI 7.95 to 21.61); and for psychiatric admissions were MH=3.62 (95% CI 2.39 to 5.49), SU=10.74 (95% CI 6.12 to 18.84) and dual diagnosis=7.74 (95% CI 4.30 to 13.94).

**Conclusions:**

Improved post-release mental health and substance use care is vital for individual and public health. Despite elevated rates of contact with community mental health and substance use services, people released from prison have disproportionately high rates of contact with emergency and unscheduled care services. This suggests that early support is either inadequate or not accessed by those in greatest need.

Policymakers and service providers should consider investment in tailored transitional and post-release intervention at individual and population level, to improve health and thus prevent later high-cost service use and avoidable mortality. Our results also suggest high-quality care must be available and accessible beyond the immediate post-release period to permit sustained engagement or engagement at a later date.

**STRENGTHS AND LIMITATIONS OF THIS STUDY**

- Nationwide population-based retrospective data linkage study with large sample size and 4-year follow-up.
- High-quality data linkage procedures to secure linkage across multiple health service, prison and deaths records.
- Examines mental health and substance use-related contacts across the care pathway: including community, outpatient, inpatient and emergency/unscheduled care services.
- Robust comparison between those released from prison and a matched general population sample without recent imprisonment.
- Administrative data are not collected for research purposes and have some limitations.

## Introduction

 The international prison population is characterised by high levels of mental health and substance use problems.^1^ Over 30 million people are released from prison globally each year,^2^ resulting in a substantial population with mental health and substance use problems returning to the community. The high prevalence of mental health and substance use problems in the prison population contributes to high rates of suicide and drug-related and alcohol-related deaths on release.^3^ Higher rates of psychiatric hospitalisation and emergency care for mental health and substance use-related conditions compared with the general population are documented up to 2 years post-release.^4 5^ High rates of death and crisis healthcare presentations related to mental health and substance use suggest early and preventative care may not be accessible, adequate or optimally utilised, thus placing considerable demand on emergency services, with implications for people released from prison and the wider population, health service delivery and public health.

Reducing drug-related harms and deaths is a public health priority in Scotland, where drug-related death rates far exceed those seen in other comparable nations, including elsewhere in the UK.^6^ Similarly, reducing alcohol-related deaths and suicides, which are high in comparison to other UK nations, is a concern.^7 8^ Contact with primary care and community-based specialist mental health and drug and alcohol services are key opportunities for preventing serious, adverse outcomes.^9^,^11^ Medication-assisted treatment has good evidence for reducing post-release drug-related deaths and increasing treatment engagement,^12^ and there is evidence for reduced drug overdoses in the 4 weeks post-release when ‘take home naloxone’ (opioid antagonist that reverses effects of opioid overdose, delivered by injection/nasal spray by anyone with training) is issued on release, including in studies conducted in Scotland.^13 14^ An Australian study identified that opioid agonist treatment reduced post-release emergency department presentations^9^ and increased engagement in mental health and primary care services.^10^ However, study time frames tend to be short, and there is evidence that sustained contact with substance use treatment is needed for long-term outcomes.^15^ Beyond opioid use, effective community-based treatment exists for other mental health and substance use disorders (including alcohol and other drugs), although there are few studies examining effectiveness with people released from prison. UK randomised controlled trials show mixed outcomes for mental health transitional interventions (delivered immediately prior to and following release). The Critical Time Intervention increased mental health service contacts for people released with serious mental illnesses compared with controls at 6 months, but the difference was not sustained at 12 months.^16^ The ENGAGER intervention for people with common mental disorders achieved at least one post-release contact for 77% of participants in the intervention, but it was not effective on the study’s primary outcome (psychological distress at 6 months post-release).^17^ To receive effective, evidence-based treatment, people need to make and *sustain* contact with relevant service providers.

Post-release contact with community mental health and substance use care is lower than levels of need identified when people are in prison. Australian cohort studies have found that 14% of people released from prison with known mental health needs were in contact with community mental healthcare at 3 months,^18^ and 25% of those with high levels of psychological distress within a year.^19^ Also in an Australian cohort study, 24% of people with substance use needs identified in prison accessed specialist services when followed up for 4 years.^20^ In a US study of people released, 26% of those self-identifying as having mental health needs and 48% self-identifying as having substance use needs received treatment in the first 3 months post-release.^21^ These low levels of contact with specialist mental health and substance use services relative to identified needs are likely to contribute to later requirements for emergency services.

There is sparse evidence for how rates and patterns of contact with community services for mental health and substance use compare between those released and demographically similar people who have not been in prison. To overcome the challenges of longitudinal follow-up post-release, teams in Australia,^22 23^ Canada^24^ and the USA^25 26^ have developed linked administrative datasets combining justice, health and deaths data to permit post-release follow-up. There are few similar studies using data from low-income and middle-income countries or Europe,^27 28^ and none from the UK. To date, studies tend to focus on one service (eg, accident and emergency) or health condition (severe mental illness), and several lack a matched comparison group. It is therefore challenging to develop a complete picture of service contact for mental health and substance use among people released from prison in comparison to the general population. Further, clarity is required regarding the extent to which current evidence is generalisable to countries with different legal and health services. Studies in different countries that examine service contacts for mental health and substance use across multiple services/settings over a longer follow-up, and with matched comparison groups, are required to develop a wider system understanding of post-release healthcare contacts for mental health and substance use, and the extent to which people who have been in prison differ from the general population.

Our objective was to compare rates of health service contact for mental health and substance use in community, outpatient, inpatient and emergency/unscheduled care services, between people released from Scottish prisons in 2015 and people who were matched on age, sex, postcode and area deprivation indices and had not recently been in prison. We addressed the following research questions:

1. To what extent are different services accessed for mental health and substance use by people released from Scottish prisons?
2. How does this compare to a general population sample matched on age, sex, postcode and postcode-derived index of deprivation and who have not recently been in prison?

## Methods

### Study design and setting

We conducted a retrospective matched cohort study using deidentified linked administrative data from the Scottish Prison Service (admissions and releases: 2010–2020), Public Health Scotland (community prescriptions, outpatient, inpatient and emergency/unscheduled care: 2010–2020) and National Records Scotland (deaths: 2015–2020). Data were used to quantify post-release mental health and substance use-related health service contacts over 4 years for all people released from Scottish prisons in 2015, compared with a matched randomly selected general population sample who had not recently been in prison. Prison admissions and release data were extracted from an operational prison population management dataset (Prisoner Records 2 (PR2)). PR2 includes all people admitted to all Scottish prisons. Data are entered by prison staff on someone’s admission to prison and updated periodically. Health service data comprised national electronic health records and prescription data. These data are systematically collected during patients’ interactions with healthcare services and entered by clinicians, call handlers, administrative staff or clinical coders. Deaths data record the date and cause of death, as recorded on the death certificate. See online supplemental file 1 for dataset details.

### Study cohort

The cohort consisted of two groups: every person released from any Scottish prison in 2015 (exposed; identified from PR2); and a comparison (unexposed) group identified from the national Community Health Index (CHI) database and cross checked against PR2 data to verify no prison exposure in the preceding 5 years (2010–2015). The CHI database includes all people in Scotland who have a unique CHI number, which is allocated at birth or at first health service contact thereafter. The index date for each exposed individual was their most recent release date in 2015. Unexposed individuals included five randomly selected individuals from the general population (without prison exposure) for each exposed individual, who were alive on the date their exposed counterpart was released from prison and matched on age (±5 years), sex, postcode and Scottish Index of Multiple Deprivation (SIMD) deciles. The SIMD is a relative measure of deprivation consisting of a ten level (1=most deprived, 10=least deprived) composite ranking of small geographic areas on seven domains: income, employment, education, health, access to services, crime and housing.^29^ The SIMD deciles were derived from the full postcode registered in the CHI database for every cohort member (exposed and unexposed). Both exposed and unexposed groups likely include people with mental health or substance needs who do not contact services, people with no mental health or substance use needs, and people whose needs have changed over time. This means we could not match on mental health or substance use needs or estimate prevalence. We instead controlled for prior mental health or substance use-related health service contact in our analysis as a proxy for needs.

### Data linkage

**Figure 1:**
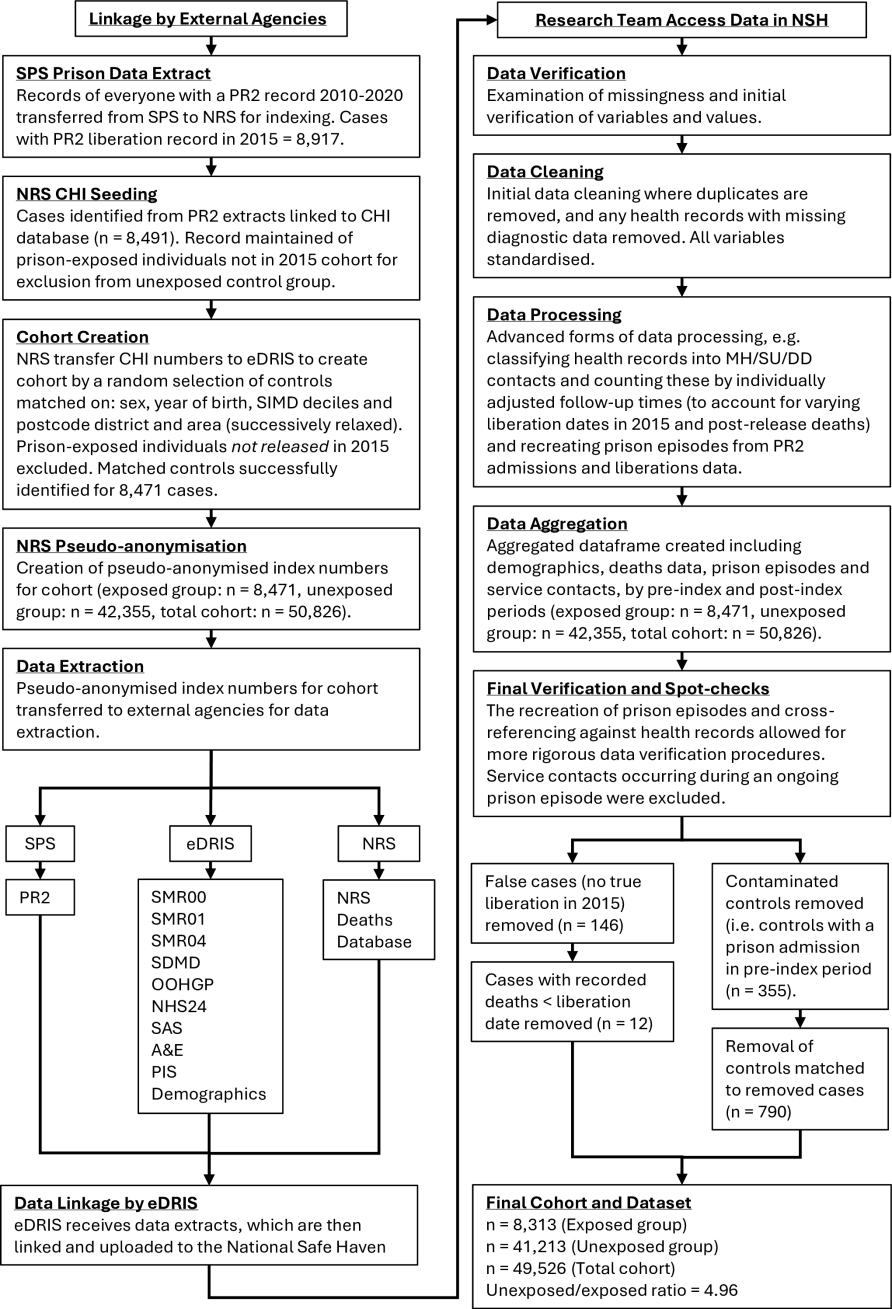
Data linkage and participant flow chart. A&E, accident and emergency database; CHI, Community Health Index; DD, dual diagnosis; eDRIS, Electronic Data Research and Innovation Service; MH, mental health; NHS, National Health Service; NHS24, 24 hour unscheduled telephone support database; NRS, National Records of Scotland; NSH, National Safe Haven; OOHGP, out-of-hours general practice database; PIS, Prescribing Information System; PR2, Prisoner Records 2; SAS, Scottish Ambulance Service database; SDMD, Scottish Drug Misuse Database; SIMD, Scottish Index of Multiple Deprivation; SMR, Scottish Morbidity Record; SMR00, outpatient attendances database; SMR01, general/acute admissions and day cases database; SMR04, psychiatric admissions and day cases database; SPS, Scottish Prison Service; SU, substance use.

Linkage was coordinated by the Electronic Data Research and Innovation Service (an independent third party; eDRIS) within Scotland’s national public health body, Public Health Scotland, with input from National Records of Scotland (NRS) for cohort creation. eDRIS conducted individual-level linkage of the cohort to records in the following health service datasets: Prescribing Information System (PIS), Scottish Morbidity Record (SMR) of outpatient attendances (SMR00), Scottish Drug Misuse Database (drug and alcohol services; SDMD), SMR01 (general/acute admissions and day cases), SMR04 (psychiatric admissions and day cases) and the emergency and unscheduled care datamart which includes ambulance (Scottish Ambulance Service (SAS)), accident and emergency (A&E), National Health Service 24-hour unscheduled telephone support (NHS24) and out-of-hours general practice (OOHGP)). The cohort was also linked to the National Records of Scotland Deaths Database (NRSDD). Community prescriptions in PIS were used as a part-proxy for primary care contacts (general practitioners provide most community prescriptions) as national general practice linkage was not possible during the study period. PIS does not include primary care contacts not resulting in a prescription. Once linked and deidentified, the data were made available for approved researchers to access within the National Safe Haven (NSH), a secure computing environment. Figure 1 shows an overview of the linkage process. See online supplemental file 1 for coding description.

### Study variables and outcomes

#### Outcomes

Our outcome was rates of contact (count of contacts per follow-up time) with health services for mental health (MH), substance use (SU) or both (‘dual diagnosis’). We categorised each health record on a mutually exclusive basis as attributable to MH, SU or dual diagnosis based on dataset coding. Where possible, International Classification of Diseases Tenth Revision (ICD-10) codes were used to determine categorisation. Datasets not using ICD-10 codes required alternative approaches. For example, contacts within mental health/psychiatric specialities recorded in SMR00 were coded as MH, and all contacts with drug and alcohol services in SDMD were coded as SU. See online supplemental file 1 for information on accessing the categorisation codes/variables.

#### Time periods

The pre-index period was the 4 years prior to, but excluding, the index date (release date, ascertained from PR2). The post-index (follow-up) period was the 4 years following, and including, the index date. Where people had more than one release date in 2015, we used the most recent. The same follow-up period was applied to the matched unexposed individuals as their associated exposed individual. We used right censoring if an exposed individual died within the 4-year follow-up, with right censoring then also applied to their matched unexposed individuals. If an unexposed individual died within the 4-year follow-up or prior to the death of their associated exposed individual, then the follow-up time was right censored for the unexposed individual alone. We used time periods, recorded deaths and PR2 data to calculate pre-index and post-index ‘time-in-community’, defined as the duration of being alive and not imprisoned, and therefore able (in theory) to access community services. All rates are calculated based on time-in-community in the post-index period.

#### Potential confounders

The principal explanatory variable was prison exposure. Time-in-community (months) in the pre-index and post-index period was calculated by reconstructing prison episodes from PR2 data. We used pre-index time-in-community as a covariate in statistical models, and the logarithm of post-index time-in-community as an offset in Poisson regression models. Previous service contacts for MH, SU or dual diagnosis were counted (accounting for time-in-community), as past contact was hypothesised to predict future contact. A count of comorbidities included in the Elixhauser Index^20^ was used to measure pre-existing comorbidity, as high levels of comorbidity (and service contact) may increase the likelihood of MH and SU-related contacts. We used a count of comorbidities as a proxy measure for general health/multimorbidity, rather than the Elixhauser Index weighted score used to predict, for example, in-hospital mortality in other studies.^30^ The Elixhauser confounder was derived from pre-index records from the SMR01 (general/acute admissions and day cases) and SMR04 (psychiatric admissions and day cases) databases.

### Study size

Study size was determined by the number of people released from Scottish prisons in 2015 (exposed) and our matching ratio of 1:5, selected to optimise statistical efficiency and enhance precision.

### Statistical methods

Once the cohort was created, anonymised and linked, researchers cleaned the data before manually reviewing and spot-checking to identify errors. Healthcare contacts recorded during a prison episode or after a recorded death were excluded. A small number of misclassified unexposed individuals who had in fact had an imprisonment episode in 2010–2015 were identified and excluded. A few exposed individuals therefore had fewer than five matched comparators. We expected very few to no missing values for any outcomes or covariates, as matching was done on the basis of complete demographic data, and outcomes were defined on the basis of records having valid data indicating contacts were associated with MH and SU. We therefore assumed that where there was no record of MH or SU-related healthcare contact, it was because that individual did not have a MH or SU-related contact in the follow-up period, rather than a contact being missing from our dataset. Covariates were operationalised and defined by the presence of valid data (time-in-community, previous service contacts, previous comorbidities).

We summarised demographic characteristics and conducted bivariate comparisons between exposed and unexposed groups to assess matching robustness and identify between-group differences in potentially confounding variables. We calculated the proportion of the exposed and unexposed groups who had at least one MH, SU or dual diagnosis contact with each service. We applied bivariate fixed-effects Poisson regression models with cluster-robust standard errors to calculate unadjusted incidence rate ratios (IRRs) for MH, SU or dual diagnosis contacts. Modelling cluster affiliation as a fixed effect accounted for the clustered data structure (each exposed individual and their five matched unexposed counterparts forming a cluster) and minimised the risk of underestimating standard errors due to cluster effects and overdispersion. To estimate adjusted IRRs (aIRRs: adjusted for pre-index MH, SU or dual diagnosis contacts; pre-index and post-index time-in-community; comorbidities), we fitted further fixed-effects Poisson models with cluster-robust standard errors. We had 28 outcome variables, including *each* contact type (MH, SU or dual diagnosis) in each service where available, and an aggregated model of contacts for *any* of MH, SU or dual diagnosis within each service. Poisson models with cluster robust standard errors were preferred over negative binomial models as they produce consistent estimates of the conditional mean in adjusted models and are robust to underdispersion or overdispersion. This allows the use of the same model specification for each combination of contact type and service.^31^ See online supplemental file 1 for model fit statistics, including mean, variance and overdispersion ratio for each outcome variable.

### Patient and public involvement

A Lived Experience Advisory Panel of people with experience of imprisonment, which varied in gender and time since release, advised the team throughout, including advising on the aims, supporting interpretation of results and informing presentation of results in this paper.

## Results

The original cohort consisted of 8917 exposed and 42 355 matched unexposed individuals. We removed 1746 individuals for linkage and data quality reasons (eg, could not be matched to the CHI database/misidentified as unexposed to imprisonment). The final cohort consisted of 8313 individuals exposed to imprisonment and 41 213 unexposed people matched from the general population (total=49 526). See figure 1 for more details.

Included individuals were predominantly male (91%) and white (96%), and on average 34.76 years old (SD=10.77) at index date. The groups were well matched on sociodemographic characteristics (table 1). The exposed group had fewer days in the community pre-index (936.81 vs 1461.00) and post-index (1074.31 vs 1391.30) and higher mean number of comorbidities (0.72 vs 0.13).

**Table 1:** Cohort characteristics: people released from prison in 2015 (exposed group) and matched individuals (unexposed group)

	Overall (N=49 526)	Exposed group (N=8313)	Unexposed group (N=41 213)
Sex			
Male	45 294 (91%)	7607 (92%)	37 687 (91%)
Female	4232 (8.5%)	706 (8.5%)	3526 (8.6%)
Urban/rural classification			
Large urban areas	21 840 (44%)	3750 (45%)	18 090 (44%)
Other urban areas	19 360 (39%)	3420 (41%)	15 940 (39%)
Accessible small towns	2940 (5.9%)	430 (5.2%)	2510 (6.1%)
Remote small towns	1080 (2.2%)	170 (2.0%)	910 (2.2%)
Very remote small towns	570 (1.2%)	110 (1.3%)	460 (1.1%)
Accessible rural areas	2710 (5.5%)	300 (3.6%)	2410 (5.8%)
Remote rural areas	660 (1.3%)	90 (1.1%)	570 (1.4%)
Very remote rural areas	340 (0.7%)	40 (0.5%)	300 (0.7%)
Missing values	20 (<0.1%)	10 (0.1%)	10 (<0.1%)
Ethnicity			
White	26 043 (96%)	8070 (97%)	17 973 (96%)
Mixed or multiple ethnic groups	99 (0.4%)	16 (0.2%)	83 (0.4%)
Asian	549 (2.0%)	129 (1.6%)	420 (2.2%)
Caribbean or black	271 (1.0%)	67 (0.8%)	204 (1.1%)
Other Ethnic group	148 (0.5%)	31 (0.4%)	117 (0.6%)
Missing values	22 416	0	22 416
SIMD quintiles			
1 (most deprived)	25 990 (52%)	4360 (52%)	21 630 (52%)
2	11 980 (24%)	2010 (24%)	9970 (24%)
3	6340 (13%)	1060 (13%)	5280 (13%)
4	4080 (8.2%)	690 (8.3%)	3390 (8.2%)
5 (least deprived)	1130 (2.3%)	180 (2.2%)	950 (2.3%)
Missing values	20 (<0.1%)	10 (0.1%)	10 (<0.1%)
Age at index date	34.76 (10.77)	34.75 (10.77)	34.76 (10.77)
Number of prison episodes			
Pre-index	0.40 (1.21)	2.41 (1.97)	0.00 (0.00)
Post-index	0.21 (0.82)	1.23 (1.64)	0.01 (0.12)
Time-in-community (days)			
Pre-index	1373.01 (264.23)	936.81 (432.80)	1461.00 (0.00)
Post-index	1338.09 (319.46)	1074.31 (458.27)	1391.30 (251.83)
Elixhauser Comorbidity Index (pre-index)	0.09 (2.18)	−0.39 (3.74)	0.18 (1.69)
Number of Elixhauser Comorbidities (pre-index)	0.23 (0.71)	0.72 (1.14)	0.13 (0.54)

Frequency (n) and percentages are shown for categorical variables. The average (M) and SD are shown for continuous variables. Index date=prison release date in 2015.

SIMD, Scottish Index of Multiple Deprivation.

Mean follow-up time was 3.83 years (SD=0.66) for the exposed group, and 3.81 (SD=0.68) for the unexposed group. Total follow-up time in person-years was 38 140.48 (exposed) and 157 181.42 (unexposed). Time-in-community in the follow-up period was 24 451.05 (exposed) and 156 986.95 (unexposed) person-years. Follow-up time varied due to death/reimprisonment. No data were missing for our outcome variables or covariates.

**Figure 2:**
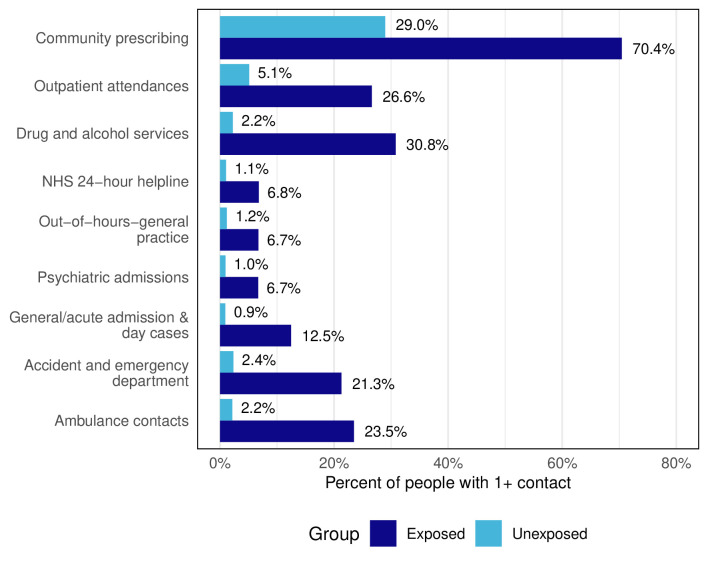
Per cent of people in each group (exposed and unexposed) with one or more service contact for mental health or substance use across services in the follow-up period.

The risk of having one or more contact(s) in each of the nine services was higher for the exposed group. Relative risk increased with the severity or urgency of issues addressed in a service. Risk ratios were lowest for community prescriptions, our proxy for primary care (2.43), substantially higher for ambulance contacts (10.68) and accident and emergency department attendance (8.88), and higher still for general/acute admissions and day cases (13.89). The risk ratio for drug and alcohol services was the highest at 14.00 (figure 2).

Across all services and reasons for contact, post-release contact rates per 1000 person-years of time-in-community were highest for MH community prescribing contacts (exposed=3382.92, unexposed=1055.04). The service and reason for contact with the lowest rate in the exposed group was MH-related general hospital admissions (1.88) and in the unexposed group was dual diagnosis-related out-of-hours general practice contacts (0.20) (table 2).

**Table 2:** Incidence rates (number of service contacts per 1000 person-years), and unadjusted and adjusted IRRs for service contacts, by service and contact type

Service	Exposed group rate (per 1000 person-years)	Unexposed group rate (per 1000 person-years)	Unadjusted IRR (95% CI)	Adjusted IRR (95% CI)
Community prescribing–Total contacts	5685.93	1207.33	4.68 (4.50 to 4.87)	1.77 (1.65 to 1.91)
Community prescribing–Mental health	3382.92	1055.04	3.16 (3.02 to 3.30)	1.80 (1.67 to 1.94)
Community prescribing–Substance use	1850.72	108.13	17.74 (16.08 to 19.57)	5.95 (4.83 to 7.32)
Community prescribing–Dual diagnosis	452.29	44.16	10.06 (8.56 to 11.83)	5.33 (3.70 to 7.68)
Outpatient attendance–Total contacts	539.12	93.93	5.89 (5.24 to 6.62)	2.61 (2.17 to 3.16)
Drug and alcohol services–Total contacts	331.89	17.99	22.01 (19.94 to 24.29)	7.13 (6.00 to 8.48)
NHS 24-hour helpline–Total contacts	66.99	6.08	12.44 (7.05 to 21.95)	7.43 (4.87 to 11.35)
NHS 24-hour helpline–Mental health	53.33	5.43	10.85 (6.05 to 19.45)	7.63 (4.93 to 11.83)
NHS 24-hour helpline–Substance use	13.66	0.65	28.15 (11.18 to 70.90)	8.29 (3.99 to 17.22)
Out-of-hours general practice–Total contacts	54.44	6.06	9.81 (5.79 to 16.62)	5.09 (3.72 to 6.95)
Out-of-hours general practice–Mental health	29.94	4.52	6.84 (3.44 to 13.58)	5.14 (3.66 to 7.22)
Out-of-hours general practice–Substance use	21.59	1.34	19.42 (13.21 to 28.57)	5.89 (3.11 to 11.14)
Out-of-hours general practice–Dual diagnosis	2.90	0.20	19.78 (8.99 to 43.53)	8.85 (2.94 to 26.63)
Psychiatric admission–Total contacts	36.28	4.49	8.45 (6.87 to 10.40)	4.99 (3.66 to 6.81)
Psychiatric admission–Mental health	17.26	2.94	6.13 (4.56 to 8.24)	3.62 (2.39 to 5.49)
Psychiatric admission–Substance use	12.11	1.04	11.87 (9.10 to 15.48)	10.74 (6.12 to 18.84)
Psychiatric admission –Dual diagnosis	6.91	0.51	15.09 (10.73 to 21.24)	7.74 (4.30 to 13.94)
General hospital admission–Total contacts	82.41	4.12	23.95 (19.47 to 29.46)	8.13 (5.22 to 12.67)
General hospital admission–Mental health	1.88	0.32	5.71 (3.48 to 9.39)	2.97 (1.43 to 6.16)
General hospital admission–Substance use	60.49	2.87	25.04 (19.62 to 31.95)	7.85 (4.42 to 13.91)
General hospital admissions–Dual diagnosis	20.04	0.94	27.35 (21.07 to 35.51)	13.11 (7.95 to 21.61)
Accident and emergency–Total contacts	191.52	11.33	20.75 (17.34 to 24.82)	6.03 (4.80 to 7.57)
Accident and emergency–Mental health	80.28	7.03	13.86 (10.97 to 17.51)	4.88 (3.78 to 6.29)
Accident and emergency–Substance use	111.24	4.31	31.96 (25.73 to 39.69)	7.98 (5.71 to 11.17)
Ambulance contact–Total contacts	196.96	9.38	26.45 (23.22 to 30.13)	7.24 (5.96 to 8.80)
Ambulance contact–Mental health	49.94	2.97	20.59 (16.74 to 25.32)	7.75 (5.76 to 10.42)
Ambulance contact–Substance use	43.76	2.22	24.69 (20.47 to 29.78)	7.58 (5.71 to 10.08)
Ambulance contact–Dual diagnosis	103.27	4.19	31.54 (26.98 to 36.86)	8.28 (6.50 to 10.55)

Incidence rates (service contacts per 1000 person-years of time-in-community), and unadjusted/adjusted IRRs by service, contact type and cohort group. Both unadjusted and adjusted IRRs are estimated from fixed effects Poisson models with cluster robust standard errors, wth ith time-in-community specified as an offset. The bivariate models only control for prison exposure. The multivariate models control for time-in-community (months) in the pre-index period (with time-in-community in the post-index period specified as an offset), pre-index service contacts, number of comorbidities and prison exposure (exposed vs unexposed). 95% CIs. Ratio=exposed group versus unexposed group.

GP, general practice; IRRs, incidence rate ratios; NHS 24, National Health Service 24-hour unscheduled telephone support.

**Figure 3:**
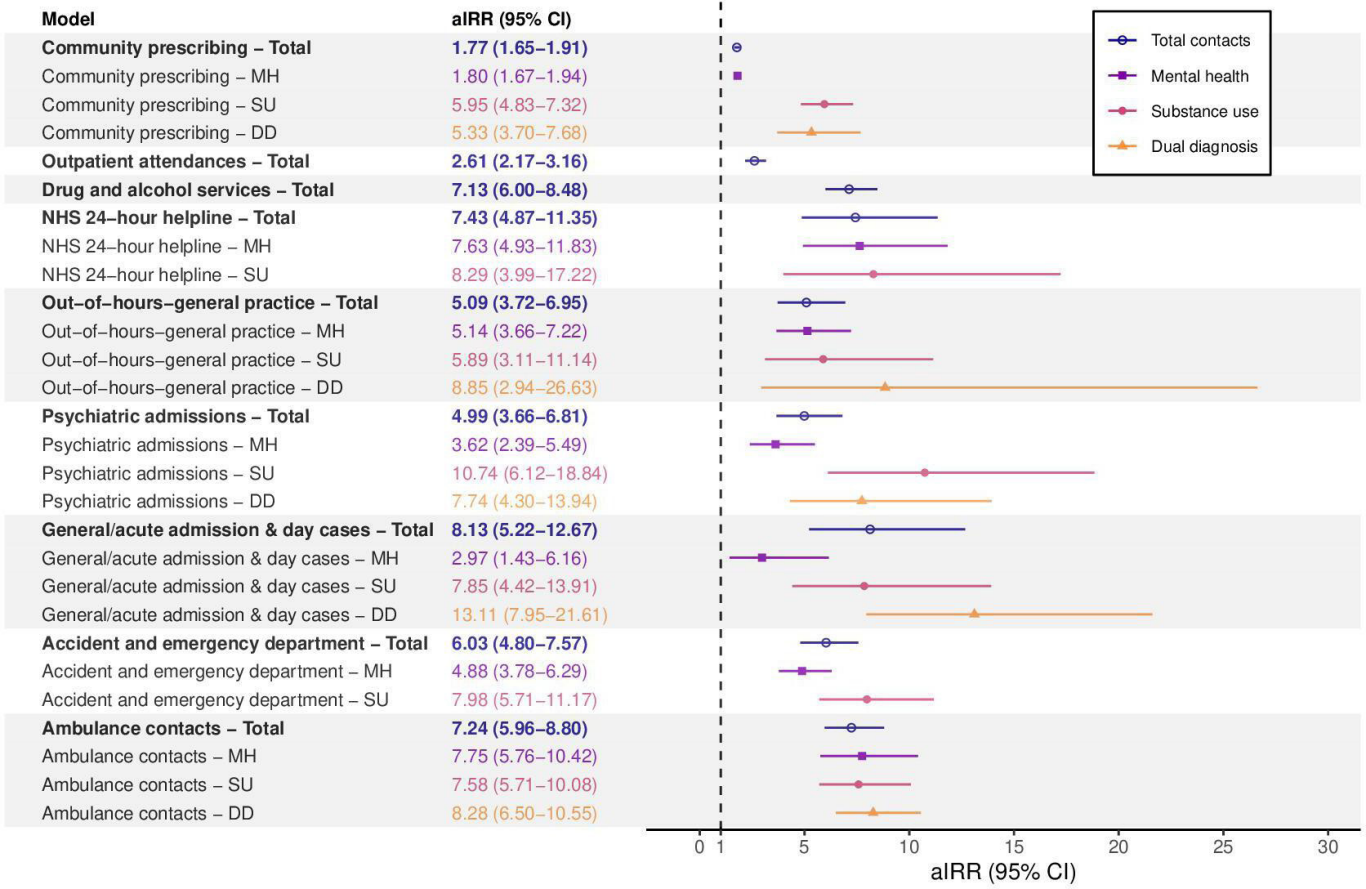
Adjusted incidence rate ratios (aIRR) with 95% CIs from 28 fixed effects Poisson models for service contacts in the follow-up period across services and disaggregated by total contacts, mental health, substance use and dual diagnosis. DD, dual diagnosis; MH, mental health; NHS, National Health Service; SU, substance use.

IRRs and aIRRs demonstrated higher rates of contact with all services and for all reasons (MH/SU/dual diagnosis) among the exposed group (see table 2 and figure 3). aIRRs for contacts for any reason (combined MH, SU and dual diagnosis) were 1.77 (95% CI 1.65 to 1.91) for community prescriptions, 2.61 (95% CI 2.17 to 3.16) for outpatient attendances, 4.99 (95% CI 3.66 to 6.81) for psychiatric admissions, 7.13 (95% CI 6.00 to 8.48) for drug and alcohol services, 8.13 (95% CI 5.22 to 12.67) for general/acute admissions and day cases, 5.09 (95% CI 3.72 to 6.95) for out-of-hours general practice, 7.43 (95% CI 4.87 to 11.35) for NHS24 calls, 6.03 (95% CI 4.80 to 7.57) for accident and emergency, and 7.24 (95% CI 5.96 to 8.80) for ambulance contacts.

aIRRs were typically higher for SU and dual diagnosis contacts compared with MH contacts, although rates of MH contacts were substantially elevated compared with the matched general population sample. CIs for MH, SU and dual diagnosis estimates typically overlapped. The aIRR for community prescriptions was 1.80 (95% CI 1.67 to 1.94) for MH, 5.95 (95% CI 4.83 to 7.32) for SU and 5.33 (95% CI 3.70 to 7.68) for dual diagnosis. aIRRs for accident and emergency department contacts were 4.88 (95% CI 3.78 to 6.29) for MH and 7.98 (95% CI 5.71 to 11.17) for SU. Ambulance contacts were the only service where aIRR point estimates for MH (7.75 (95% CI 5.76 to 10.42)) were higher than SU (7.58 (95% CI 5.71 to 10.08)) and dual diagnosis (8.28 (95% CI 6.50 to 10.55)).

## Discussion

We present an internationally original analysis concentrating on post-release mental health and substance use-related healthcare contacts across community, outpatient, inpatient and emergency/unscheduled healthcare services. We compared people released from prison with a matched general population group who had not recently been in prison. Our results make a significant contribution, as the first analysis of post-release mental health and substance use-related service contacts that takes a wide health system perspective, uses UK data and has a matched general population comparison group.

Rates of healthcare contacts for mental health, substance use and dual diagnosis are elevated among people released from prison compared with a randomly selected, demographically matched (age, sex, postcode and SIMD) group that had not been imprisoned in the preceding five years. Differences were substantial, despite matching on sex, age and indices of deprivation and controlling for prior mental health and substance use-related healthcare contacts, time-in-community and comorbidities. Differences in contact rates between exposed and unexposed groups widened as services became focused on more severe and urgent needs, ie, from community prescriptions to inpatient admissions and ambulance contacts.

Higher rates of service contact compared with the general population and matched comparators are reported in studies from other high-income countries. These typically focus on primary and emergency care and are predominantly conducted in North America.^5 24 32 33^ Our findings confirm substantially higher rates of service contact in a European context and add to the international literature by focusing on contacts for mental health and substance use specifically and across a wide range of health services. Our use of a matched general population comparator who had not been in prison extends international evidence that shows rates of post-release mental health and substance use-related health service contacts are below levels of need identified in custody.^18^,^20^ Despite elevated rates of contact with community and outpatient services seen in our cohort, the international literature suggests there are likely many people released who need support but do not contact services until crisis point, contributing to high levels of contact with emergency or unscheduled care (in our study consisting of contact with accident and emergency department, ambulance, out-of-hours general practice and NHS 24 hr unscheduled telephone support services). It is a public health priority that these individuals are identified and supported to engage with high-quality support that reduces later need for costly emergency services.

Differences in rates of contact between those who had, and had not, been released from prison (aIRRs) were larger for substance use compared with mental health for psychiatric inpatient admissions, general/acute inpatient admissions and accident and emergency department attendance. Differences in rates for dual diagnosis contacts were typically higher than for substance use contacts. However, this disparity was not evident for ambulance contacts or NHS24 (emergency health support not requiring an ambulance) calls, where contact rates for people released from prison were eight times higher than in the matched comparator group for mental health, substance use and dual diagnosis. This suggests substance use potentially has a greater impact on people who have been in prison than similar people without prison exposure, resulting in a greater need for acute and emergency services (potentially contributed to by associated physical health complications). Rates of contact with community prescribers and outpatient services are also larger for substance use than mental health when compared with the matched group. This must be interpreted while acknowledging that the raw number of contacts for substance use prescriptions and with drug and alcohol services is lower than the number of contacts for mental health prescriptions and outpatient mental healthcare. This should also be seen in the context of Scottish services. Mental health services in Scotland are complicated and face sustained resourcing issues, contributing to long waiting lists and strict engagement requirements, often including being substance-free.^34 35^ This may lead to the mental health needs among people who use drugs and alcohol going unmet, and a resultant increase in substance use to manage symptoms or distress. Higher contact rates with community prescribers for substance use among people released compared with the matched general population sample, and a large proportion of people released in contact with drug and alcohol services, suggest efforts to sustain or initiate post-release opioid agonist prescriptions and substance use treatment may be more successful than efforts to sustain or initiate mental health treatment. The relative inaccessibility of mental health support could explain why absolute rates of contact drop substantially between community prescribing and outpatient mental healthcare, and that despite very high levels of known mental ill-health in the prison population, aIRRs for mental health outpatient attendances were 2.6, compared with 7.1 for drug and alcohol services. However, the absolute rates of contact between prescribing and drug and alcohol services also see a substantial drop. Together, these results suggest mental health and substance use needs among people released from prison, beyond general practice prescribing, may not be being met and consequently needs may escalate until requiring emergency care.

The reasons for elevated rates of service contacts require examination. They may reflect a higher prevalence of mental health and substance use problems in the prison population.^1^ However, by controlling for other predictors of contacts, including prior mental health and substance use-related contacts, our results suggest imprisonment and release may make specific contributions to post-release service contact rates. This could be explained by escalating mental health and substance use problems during imprisonment and on release.^36^,^38^ Alternatively, increased contact rates with community prescribers and outpatient services could imply that prison has a facilitative effect on people contacting services post-release. This could be due to experiencing a relatively stable period in prison that allows for self-reflection, prison staff identifying needs or making contact with support services/workers while in prison. People receiving mental health and substance use care in prison may be linked to services on release.^38^ Evidence suggests that pre-release contact with services for severe mental illness and opioid dependence may increase post-release contact with specialist services, at least in the short term.^16 21 39^ However, care continuity at scale for wider mental health and substance use needs is rarely achieved, often leaving people attempting to navigate complicated, under-resourced community services, while balancing competing priorities which traditional services struggle to accommodate.^3440^,^42^ In our results, the elevated emergency services contact rates, paired with international evidence about challenges in post-release care continuity, would suggest that increased mental health and substance use needs following imprisonment and release may be the more plausible explanation for increased contact rates. Research, including qualitative studies, is needed to determine the drivers of higher rates of post-release service contacts in different services.

Regardless of higher rates of contact for mental health and substance use with community prescribers and outpatient services, large disparities remain in contact rates for inpatient and emergency services. This suggests that either primary care and community service contacts do not result in adequate mental health and substance use care for this population (eg, due to the need to manage more complex needs and presentations, operating under resource constraints or with unsuitable models of care), or that many people released with mental health and substance use problems are not contacting any services until they are at a crisis point. Concerningly, over 1 in 5 (21.3%) of people released from prison had at least one accident and emergency department presentation for mental health or substance use, and around a quarter (23.5%) had at least one ambulance contact. A similar proportion of a US cohort had at least one emergency department contact in the year post-release, with mental health and substance use significantly more likely to be the reason for attendance among those released from prison compared with the general population.^4^ At 2 years, people released in Canada had emergency department contact rates for any condition at 3.4 (men) and 4.7 (women) times their matched comparison group.^5^ In our 4-year follow-up, where we might expect rates to equalise as someone resettles in the community, we found higher accident and emergency department contact rates for mental health (4.9) and substance use (8.0) compared with the matched group. These findings reflect US evidence that mental health and substance use likely drive differences in accident and emergency department attendance rates,^4^ which may be obscured in the Canadian data where all conditions are considered together. Our longer follow-up suggests that need likely continues well beyond the immediate release period. Qualitative studies are needed to understand why these patterns are observed, explore post-release experiences of care and evaluate whether current models are appropriate for the complex needs in this population. Together, this could inform decisions about optimal intervention points for individual and public health interventions that reduce the need for costly emergency services.

Our study has several strengths, including: using a nationwide cohort and a whole population sample from which to identify a matched (unexposed) group; a large sample size (nearly 50 000); 4-year follow-up; high-quality data linkage procedures; and linkage across multiple health services, spanning community to emergency care. It provides the basis for monitoring trends and informing policy and practice developments to optimise individual outcomes and improve public health. Our research has two main limitations. First, linked administrative data are not collected for research and have some constraints. We reconstructed prison episodes (to establish time-in-community and remove contacts during prison episodes) from PR2. PR2 contained some inconsistencies in recorded dates of admission and release. We adapted a bespoke algorithm, developed at Public Health Scotland,^43^ which is the best available approach for working with PR2. There may be variation in recording practices (eg, diagnostic information recorded/omitted). Including all prisons and health boards in Scotland mitigates some impact of practice variation. Given high comorbidity in the prison population^44^ and the low number of contacts recorded as dual diagnosis (table 1), it is likely many people had contact with services for both needs, but only one was recorded (or disclosed). It is therefore important not to conflate service contacts with prevalence rates. A larger proportion of contacts coded as dual diagnosis may alter the sub-patterning of results (between mental health, substance use and dual diagnosis), but overall results for contacts for any of mental health, substance use or dual diagnosis would be unchanged. Some datasets did not use ICD-10 codes and required alternative coding. There may be other ways to categorise the data; however, the overall pattern of our results is clear. We used community prescription contacts as a part-proxy for primary care, as nationwide primary care data were unavailable. Future research will benefit from linkage to a national general practice database to identify all primary care contacts. Using linked administrative data limits selection of confounding variables to those available in the dataset. There may have been additional confounding variables that we could not capture, such as whether mental health or substance use needs were present at the index date. Second, our study was conducted in one country. Our cohort is typical of prison cohorts internationally, but caution should be taken when generalising findings to countries with different health/justice systems and service models; attitudes towards mental health, substance use and crime; and sociodemographic features.

## Conclusions

This study demonstrates clearly that contact rates with health services for mental health and substance use are elevated among people released from prison. Despite higher contact rates with community and outpatient services, emergency and unscheduled care contact rates were starkly elevated, and a strikingly high proportion of those released from prison used ambulance services and accident and emergency department care.

These results present the first system-wide view of post-release mental health and substance use-related healthcare contacts, comparing people released from prison to a matched group unexposed to imprisonment. The study thus makes a significant original contribution to understanding mental health and substance use-related service contact following a period in prison that can inform health, justice and social care policy.

Public health attention is needed to address the mental health and substance use needs of people released from prison, before they reach a crisis point requiring costly emergency care.

### Implications for policy, practice and research

Policymakers across health and justice should consider individual and population level interventions to address the increased level of need for mental health and substance use support on release from prison, to prevent later high-cost service use and avoidable mortality, and avoid increased pressures on stretched health services.

The importance of continuity of care on release is well documented. It is vital that people receiving mental health or substance use treatment in prison are linked to community provision for continued treatment (including opioid agonist treatment) and support. However, this relies on (1) those with mental health and substance use needs being identified and receiving care in prison that can be continued and (2) community services having capacity to receive new patients in a timely fashion. Signposting or attempts to link to services with substantial waiting lists is unlikely to be beneficial. Further, our results indicate a need to consider care provision beyond the immediate post-release period so that people have the opportunity to sustain engagement with high quality support for as long as necessary, or to engage later post-release.

Primary care, community mental health and community drug and alcohol services must be resourced to respond to the needs of people released from prison. Where services operating traditional models struggle to meet the complex needs of people living with intersecting social disadvantage, access to ‘Inclusion Health’ interventions and services on release may be useful. These services and interventions require a flexible and non-stigmatising workforce with the skills to address multi-morbidity and the social determinants of health in an integrated way.^45^ Encouraging and resourcing services to apply a ‘missingness lens’ may also be beneficial. A missingness lens involves deliberate action to identify and respond to missed appointments, acknowledging that these are a risk indicator for adverse outcomes.^46^ However, evidence of the effectiveness of these services/approaches is still developing.

Research is needed to better understand the reasons behind elevated post-release mental health and substance use contact patterns in different services; identify whether particular groups of people released may be at risk of unmet needs for different conditions at different times; and to establish feasible and effective ways to intervene to improve mental health and substance use outcomes for people released from prison, particularly to reduce the need for costly emergency care and avoidable mortality. Research should take account of contextual variation in law, practice across health, justice and social care services/systems, and consider what works in the immediate transitional period and over the longer term.

## Supplementary material

10.1136/bmjopen-2025-107586 online supplemental file 1

## Data Availability

No data are available.
