# Supplementary material for: Health service contacts for mental health and substance use on release from prison: a retrospective population-based data linkage study
Source: BMJ Open. 2026 Feb 4;16(2):e107586. doi: 10.1136/bmjopen-2025-107586 (PMC12878195; doi:10.1136/bmjopen-2025-107586)
Supplement: online supplemental file 1 [file bmjopen-16-2-s001.docx]

Supplementary material

Contents

[Datasets 2](#_Toc199231489)

[Data linkage process 3](#_Toc199231490)

[Codes used to categorise contacts as mental health or substance use 4](#_Toc199231491)

[Model fit statistics 5](#_Toc199231492)

[Extended model outputs 5](#_Toc199231493)

# Datasets

**Table S1: Datasets used in the study**

| Dataset | Description | Purpose |
| --- | --- | --- |
| The Community Health Index (CHI) | A population register used in Scotland for health care purposes. The CHI number uniquely identifies a person on the index and contains basic demographic data. CHI number is allocated at birth/first healthcare contact thereafter. | Unique identifier for each person in the cohort which facilitates linkage to all other datasets. |
| Outpatient Appointments and Attendances (SMR00) | Episode-level data from patients on new and follow-up appointments at outpatient clinics in all specialities (except A&E and Genito-Urinary Medicine). | Derive service contacts (outcome) and pre-index health status (covariate). |
| The General / Acute and Inpatient Day Case dataset (SMR01) | Episode-level data on hospital inpatient and day case discharges from acute specialities from hospitals in Scotland including diagnosis. The dataset contains episode management data. |  |
| The Mental Health Inpatient and Day Case dataset (SMR04) | Episode-level data on patients receiving care at psychiatric hospitals, including diagnosis, at the point of admission and discharge. |  |
| Scottish Drug Misuse Database (SDMD) | Information about patients seen at specialist drug and alcohol services, general practitioners and prison drug services. This includes prescription profile, and initial and follow-up information |  |
| Scotland’s telehealth service (NHS24) | Information about unscheduled care contacts with Scotland’s national telehealth and telecare service, including information on the nature and outcome of the call. |  |
| Scottish Ambulance Service (SAS) | Episode-level data on patients who have used unscheduled emergency transportation. Contains the Advanced Medical Priority Dispatch System (AMPDS) code and information on substance use. |  |
| Primary Care Out of Hours (OOH) | Episode-level data on patients receiving out of hours primary care services. Contains Read codes. |  |
| Accident and Emergency (A&E) | Episode-level data on patients receiving unscheduled care across emergency departments, minor injuries units and community units. Contains ICD10 codes and diagnostic group codes. |  |
| Prescribing Information System (PIS) | Records all medicines prescribed and dispensed in the community, and prescriptions written in hospitals that are dispensed in the community. As GPs write the vast majority of these (a small minority are written by other authorised prescribers e.g., nurses, dentists), we use prescribing data as a proxy for accessing primary care for MH/SU. Contains British National Formulary (BNF) codes, used to identify prescriptions issued for MH/SU-related medicines. |  |
| National Register of Scotland Death Records (NRSD) | Database containing information on all registered deaths in Scotland with information on cause(s) of deaths. | Combined with prison records to calculate ‘time-in-community’ (i.e. time in the post-index period when the individual could access community-based services, which was used as a denominator when adjusting service contacts for time-in-community), and for descriptive statistics on mortality rates. |
| Prisoner Records 2 (PR2) | Prison admission and liberation records and demographic data for all people in Scottish Prison Service (SPS) establishments. The dataset is split into two files – admissions and liberations, with demographic data associated with the liberations dataset. | Used to reconstruct prison episodes (distinct period where the person was in prison), which was used as a covariate (time-in-community), and for adjusting service contacts (outcomes) for time-in-community. |

# Codes used to categorise contacts as mental health or substance use

Databases contained diffferent variables for recording reasons for a contact. For each database, we identified the variables where relevant codes were recorded, then identified the codes that were indicative of a mental health (MH) or substance use(SU) related contact. The table below highlights the variables used for categorisation in each of the nine datasets, and whether we were able to identify MH, SU or dual diagnosis (DD) contacts using the codes recorded. Due to volume of codes, we have compiled these in an Excel spreadsheet accessible on our GitHub stie: <https://github.com/rkjellgren/RELEASE>

A GitHub account is free and you can follow the ongoing work of the team.

**Table S2: Codes used to categorise contacts as mental health or substance use**

| **Dataset** | **Classification variables** | **Delineation** |
| --- | --- | --- |
| SMR00 | Clinical speciality of service | MH |
| SMR01 | ICD10 diagnoses | MH, SU, DD |
| SMR04 | ICD10 diagnoses | MH, SU, DD |
| SDMD | (all contacts SU) | SU |
| NHS24 | Symptoms reported | MH, SU |
| SAS | AMPDS codes, complaint codes, substance use-specific variables | MH, SU, DD |
| OOH | Read codes | MH, SU, DD |
| A&E | ICD10 diagnoses, diagnostic group codes, intent of injury | MH, SU |
| PIS | BNF codes of prescribed items | MH, SU, DD |

# Model fit statistics

**Table S3: Model fit statistics for 28 fixed effects Poisson models with clustered standard errors.**

| **Model** | **n** | **Log-likelihood** | **AIC** | **BIC** | **Pseudo-R2** | **Multicollinearity** | **Dispersion Ratio** | **Outcome Mean** | **Outcome Variance** |
| --- | --- | --- | --- | --- | --- | --- | --- | --- | --- |
| Community prescribing (PIS) - Total contacts | 49526 | -220957.27 | 458548.54 | 531823.41 | 0.61 | VIF<5 | 14.62 | 6.63 | 244.92 |
| Community prescribing (PIS) - MH | 49526 | -194690.79 | 406015.58 | 479290.45 | 0.56 | VIF<5 | 16.26 | 5.01 | 154.50 |
| Community prescribing (PIS) - SU | 49526 | -33482.41 | 83598.82 | 156873.70 | 0.83 | VIF<5 | 17.96 | 1.26 | 36.24 |
| Community prescribing (PIS) - DD | 49526 | -12700.08 | 42034.16 | 115309.04 | 0.83 | VIF<5 | 5.05 | 0.36 | 10.24 |
| Outpatient contacts (SMR00) - Total contacts | 49526 | -33510.62 | 83655.24 | 156930.11 | 0.63 | VIF<5 | 60010325.72 | 0.56 | 12.67 |
| Specialist addiction services (SDMD) - Total contacts | 49526 | -11197.90 | 39029.81 | 112304.68 | 0.69 | VIF<5 | 3.05 | 0.22 | 1.04 |
| NHS 24-hour helpline (NHS24) - Total contacts | 49526 | -3000.97 | 22635.94 | 95910.81 | 0.79 | VIF<5 | 2328821027.19 | 0.05 | 2.22 |
| NHS 24-hour helpline (NHS24) - MH | 49526 | -2679.82 | 21993.64 | 95268.51 | 0.77 | VIF<5 | 2680456348.21 | 0.04 | 1.28 |
| NHS 24-hour helpline (NHS24) - SU | 49526 | -422.29 | 17478.58 | 90753.46 | 0.87 | VIF<5 | 0.03 | 0.01 | 0.56 |
| Out-of-hours GP (OOH) - Total contacts | 49526 | -3227.09 | 23088.18 | 96363.06 | 0.73 | VIF<5 | 5313.46 | 0.05 | 1.21 |
| Out-of-hours GP (OOH) - MH | 49526 | -2168.07 | 20970.15 | 94245.02 | 0.74 | VIF<5 | 1.89 | 0.03 | 0.94 |
| Out-of-hours GP (OOH) - SU | 49526 | -1011.69 | 18657.37 | 91932.25 | 0.77 | VIF<5 | 0.34 | 0.01 | 0.12 |
| Out-of-hours GP (OOH) - DD | 49526 | -194.65 | 17023.31 | 90298.18 | 0.75 | VIF<5 | 0.01 | 0.00 | 0.00 |
| Psychiatric hospital admissions (SMR04) - Total contacts | 49526 | -2619.72 | 21873.44 | 95148.31 | 0.66 | VIF<5 | 4.49 | 0.03 | 0.11 |
| Psychiatric hospital admissions (SMR04) - MH | 49526 | -1519.20 | 19672.40 | 92947.27 | 0.69 | VIF<5 | 148.92 | 0.02 | 0.07 |
| Psychiatric hospital admissions (SMR04) - SU | 49526 | -774.62 | 18183.24 | 91458.11 | 0.72 | VIF<5 | 0.06 | 0.01 | 0.02 |
| Psychiatric hospital admissions (SMR04) - DD | 49526 | -443.03 | 17520.06 | 90794.93 | 0.72 | VIF<5 | 0.20 | 0.01 | 0.01 |
| General hospital admissions (SMR01) - Total contacts | 49526 | -3336.98 | 23307.96 | 96582.84 | 0.72 | VIF<5 | 0.63 | 0.05 | 0.27 |
| General hospital admissions (SMR01) - MH | 49526 | -204.78 | 17043.55 | 90318.43 | 0.71 | VIF<5 | 0.01 | 0.00 | 0.00 |
| General hospital admissions (SMR01) - SU | 49526 | -2405.22 | 21444.43 | 94719.31 | 0.74 | VIF<5 | 0.70 | 0.04 | 0.19 |
| General hospital admissions (SMR01) - DD | 49526 | -947.45 | 18528.90 | 91803.77 | 0.73 | VIF<5 | 0.10 | 0.01 | 0.02 |
| Accident and emergency department (A&E) - Total contacts | 49526 | -8140.68 | 32915.36 | 106190.23 | 0.68 | VIF<5 | 6245.60 | 0.13 | 1.14 |
| Accident and emergency department (A&E) - MH | 49526 | -4865.43 | 26364.87 | 99639.74 | 0.64 | VIF<5 | 131971.42 | 0.06 | 0.41 |
| Accident and emergency department (A&E) - SU | 49526 | -3882.75 | 24399.50 | 97674.38 | 0.75 | VIF<5 | 0.86 | 0.07 | 0.58 |
| Ambulance call-outs (SAS) - Total contacts | 49526 | -7412.27 | 31458.55 | 104733.42 | 0.69 | VIF<5 | 162282.12 | 0.13 | 0.74 |
| Ambulance call-outs (SAS) - MH | 49526 | -2416.77 | 21467.53 | 94742.41 | 0.71 | VIF<5 | 0.69 | 0.03 | 0.13 |
| Ambulance call-outs (SAS) - SU | 49526 | -2078.15 | 20790.30 | 94065.18 | 0.70 | VIF<5 | 594.30 | 0.03 | 0.06 |
| Ambulance call-outs (SAS) - DD | 49526 | -4057.05 | 24748.10 | 98022.98 | 0.70 | VIF<5 | 5.03 | 0.06 | 0.24 |

# Extended model outputs

**Table S4: Model estimates for all covariates for 28 fixed effects Poisson models with clustered standard errors**

| **Model** | **Term** | **IRR (95% CI)** | **P-Value** |
| --- | --- | --- | --- |
| Community prescribing (PIS) - Total contacts | Exposed vs. Unexposed | 1.77 (1.65-1.91) | 0.00 |
| Community prescribing (PIS) - Total contacts | (n) Pre-index Contacts | 1.05 (1.05-1.05) | 0.00 |
| Community prescribing (PIS) - Total contacts | (n) Pre-index Months in Community | 0.96 (0.96-0.97) | 0.00 |
| Community prescribing (PIS) - Total contacts | (n) Comorbidities | 1.16 (1.13-1.19) | 0.00 |
| Community prescribing (PIS) - MH | Exposed vs. Unexposed | 1.8 (1.67-1.94) | 0.00 |
| Community prescribing (PIS) - MH | (n) Pre-index Contacts | 1.06 (1.06-1.06) | 0.00 |
| Community prescribing (PIS) - MH | (n) Pre-index Months in Community | 0.97 (0.97-0.98) | 0.00 |
| Community prescribing (PIS) - MH | (n) Comorbidities | 1.16 (1.13-1.2) | 0.00 |
| Community prescribing (PIS) - SU | Exposed vs. Unexposed | 5.95 (4.83-7.32) | 0.00 |
| Community prescribing (PIS) - SU | (n) Pre-index Contacts | 1.13 (1.12-1.14) | 0.00 |
| Community prescribing (PIS) - SU | (n) Pre-index Months in Community | 0.96 (0.95-0.96) | 0.00 |
| Community prescribing (PIS) - SU | (n) Comorbidities | 1.44 (1.31-1.6) | 0.00 |
| Community prescribing (PIS) - DD | Exposed vs. Unexposed | 5.33 (3.7-7.68) | 0.00 |
| Community prescribing (PIS) - DD | (n) Pre-index Contacts | 1.17 (1.13-1.22) | 0.00 |
| Community prescribing (PIS) - DD | (n) Pre-index Months in Community | 0.96 (0.94-0.97) | 0.00 |
| Community prescribing (PIS) - DD | (n) Comorbidities | 1.34 (1.13-1.58) | 0.00 |
| Outpatient contacts (SMR00) - Total contacts | Exposed vs. Unexposed | 2.61 (2.17-3.16) | 0.00 |
| Outpatient contacts (SMR00) - Total contacts | (n) Pre-index Contacts | 1.15 (1.1-1.2) | 0.00 |
| Outpatient contacts (SMR00) - Total contacts | (n) Pre-index Months in Community | 0.98 (0.97-0.98) | 0.00 |
| Outpatient contacts (SMR00) - Total contacts | (n) Comorbidities | 1.52 (1.4-1.66) | 0.00 |
| Specialist addiction services (SDMD) - Total contacts | Exposed vs. Unexposed | 7.13 (6-8.48) | 0.00 |
| Specialist addiction services (SDMD) - Total contacts | (n) Pre-index Contacts | 1.97 (1.82-2.13) | 0.00 |
| Specialist addiction services (SDMD) - Total contacts | (n) Pre-index Months in Community | 0.97 (0.96-0.98) | 0.00 |
| Specialist addiction services (SDMD) - Total contacts | (n) Comorbidities | 1.46 (1.33-1.59) | 0.00 |
| NHS 24-hour helpline (NHS24) - Total contacts | Exposed vs. Unexposed | 7.43 (4.87-11.35) | 0.00 |
| NHS 24-hour helpline (NHS24) - Total contacts | (n) Pre-index Contacts | 2.44 (1.6-3.73) | 0.00 |
| NHS 24-hour helpline (NHS24) - Total contacts | (n) Pre-index Months in Community | 1 (0.99-1.02) | 0.59 |
| NHS 24-hour helpline (NHS24) - Total contacts | (n) Comorbidities | 1.94 (1.61-2.33) | 0.00 |
| NHS 24-hour helpline (NHS24) - MH | Exposed vs. Unexposed | 7.63 (4.93-11.83) | 0.00 |
| NHS 24-hour helpline (NHS24) - MH | (n) Pre-index Contacts | 2.52 (1.53-4.15) | 0.00 |
| NHS 24-hour helpline (NHS24) - MH | (n) Pre-index Months in Community | 1.01 (0.99-1.02) | 0.55 |
| NHS 24-hour helpline (NHS24) - MH | (n) Comorbidities | 1.85 (1.53-2.22) | 0.00 |
| NHS 24-hour helpline (NHS24) - SU | Exposed vs. Unexposed | 8.29 (3.99-17.22) | 0.00 |
| NHS 24-hour helpline (NHS24) - SU | (n) Pre-index Contacts | 24.03 (7.75-74.53) | 0.00 |
| NHS 24-hour helpline (NHS24) - SU | (n) Pre-index Months in Community | 1.01 (0.99-1.04) | 0.32 |
| NHS 24-hour helpline (NHS24) - SU | (n) Comorbidities | 3.24 (2.37-4.43) | 0.00 |
| Out-of-hours GP (OOH) - Total contacts | Exposed vs. Unexposed | 5.09 (3.72-6.95) | 0.00 |
| Out-of-hours GP (OOH) - Total contacts | (n) Pre-index Contacts | 8.39 (4.44-15.86) | 0.00 |
| Out-of-hours GP (OOH) - Total contacts | (n) Pre-index Months in Community | 0.99 (0.97-1.01) | 0.23 |
| Out-of-hours GP (OOH) - Total contacts | (n) Comorbidities | 1.91 (1.64-2.22) | 0.00 |
| Out-of-hours GP (OOH) - MH | Exposed vs. Unexposed | 5.14 (3.66-7.22) | 0.00 |
| Out-of-hours GP (OOH) - MH | (n) Pre-index Contacts | 10.13 (4.14-24.79) | 0.00 |
| Out-of-hours GP (OOH) - MH | (n) Pre-index Months in Community | 1 (0.99-1.02) | 0.76 |
| Out-of-hours GP (OOH) - MH | (n) Comorbidities | 1.91 (1.64-2.22) | 0.00 |
| Out-of-hours GP (OOH) - SU | Exposed vs. Unexposed | 5.89 (3.11-11.14) | 0.00 |
| Out-of-hours GP (OOH) - SU | (n) Pre-index Contacts | 12.6 (4.64-34.24) | 0.00 |
| Out-of-hours GP (OOH) - SU | (n) Pre-index Months in Community | 0.98 (0.95-1.01) | 0.20 |
| Out-of-hours GP (OOH) - SU | (n) Comorbidities | 2.23 (1.78-2.79) | 0.00 |
| Out-of-hours GP (OOH) - DD | Exposed vs. Unexposed | 8.85 (2.94-26.63) | 0.00 |
| Out-of-hours GP (OOH) - DD | (n) Pre-index Contacts | 3446241026.33 (2620276389.93-4532566586.19) | 0.00 |
| Out-of-hours GP (OOH) - DD | (n) Pre-index Months in Community | 0.97 (0.92-1.03) | 0.33 |
| Out-of-hours GP (OOH) - DD | (n) Comorbidities | 1.39 (0.99-1.95) | 0.05 |
| Psychiatric hospital admissions (SMR04) - Total contacts | Exposed vs. Unexposed | 4.99 (3.66-6.81) | 0.00 |
| Psychiatric hospital admissions (SMR04) - Total contacts | (n) Pre-index Contacts | 5.51 (3.78-8.04) | 0.00 |
| Psychiatric hospital admissions (SMR04) - Total contacts | (n) Pre-index Months in Community | 0.99 (0.97-1) | 0.06 |
| Psychiatric hospital admissions (SMR04) - Total contacts | (n) Comorbidities | 1.66 (1.43-1.91) | 0.00 |
| Psychiatric hospital admissions (SMR04) - MH | Exposed vs. Unexposed | 3.62 (2.39-5.49) | 0.00 |
| Psychiatric hospital admissions (SMR04) - MH | (n) Pre-index Contacts | 9.57 (4.41-20.78) | 0.00 |
| Psychiatric hospital admissions (SMR04) - MH | (n) Pre-index Months in Community | 0.98 (0.96-0.99) | 0.01 |
| Psychiatric hospital admissions (SMR04) - MH | (n) Comorbidities | 1.48 (1.21-1.82) | 0.00 |
| Psychiatric hospital admissions (SMR04) - SU | Exposed vs. Unexposed | 10.74 (6.12-18.84) | 0.00 |
| Psychiatric hospital admissions (SMR04) - SU | (n) Pre-index Contacts | 6.29 (3.79-10.45) | 0.00 |
| Psychiatric hospital admissions (SMR04) - SU | (n) Pre-index Months in Community | 1.02 (0.99-1.04) | 0.21 |
| Psychiatric hospital admissions (SMR04) - SU | (n) Comorbidities | 2.25 (1.81-2.81) | 0.00 |
| Psychiatric hospital admissions (SMR04) - DD | Exposed vs. Unexposed | 7.74 (4.3-13.94) | 0.00 |
| Psychiatric hospital admissions (SMR04) - DD | (n) Pre-index Contacts | 22.44 (2.72-185.27) | 0.00 |
| Psychiatric hospital admissions (SMR04) - DD | (n) Pre-index Months in Community | 0.99 (0.97-1.02) | 0.51 |
| Psychiatric hospital admissions (SMR04) - DD | (n) Comorbidities | 2.05 (1.55-2.72) | 0.00 |
| General hospital admissions (SMR01) - Total contacts | Exposed vs. Unexposed | 8.13 (5.22-12.67) | 0.00 |
| General hospital admissions (SMR01) - Total contacts | (n) Pre-index Contacts | 2.24 (1.65-3.04) | 0.00 |
| General hospital admissions (SMR01) - Total contacts | (n) Pre-index Months in Community | 0.98 (0.96-1) | 0.01 |
| General hospital admissions (SMR01) - Total contacts | (n) Comorbidities | 2.54 (2.2-2.93) | 0.00 |
| General hospital admissions (SMR01) - MH | Exposed vs. Unexposed | 2.97 (1.43-6.16) | 0.00 |
| General hospital admissions (SMR01) - MH | (n) Pre-index Contacts | 157081548.9 (60772557.87-406015706.24) | 0.00 |
| General hospital admissions (SMR01) - MH | (n) Pre-index Months in Community | 0.98 (0.94-1.02) | 0.30 |
| General hospital admissions (SMR01) - MH | (n) Comorbidities | 2.12 (1.62-2.77) | 0.00 |
| General hospital admissions (SMR01) - SU | Exposed vs. Unexposed | 7.85 (4.42-13.91) | 0.00 |
| General hospital admissions (SMR01) - SU | (n) Pre-index Contacts | 2.08 (1.26-3.43) | 0.00 |
| General hospital admissions (SMR01) - SU | (n) Pre-index Months in Community | 0.98 (0.96-1) | 0.04 |
| General hospital admissions (SMR01) - SU | (n) Comorbidities | 2.69 (2.27-3.19) | 0.00 |
| General hospital admissions (SMR01) - DD | Exposed vs. Unexposed | 13.11 (7.95-21.61) | 0.00 |
| General hospital admissions (SMR01) - DD | (n) Pre-index Contacts | 7.6 (2.97-19.4) | 0.00 |
| General hospital admissions (SMR01) - DD | (n) Pre-index Months in Community | 0.99 (0.97-1.01) | 0.32 |
| General hospital admissions (SMR01) - DD | (n) Comorbidities | 2.64 (2.04-3.42) | 0.00 |
| Accident and emergency department (A&E) - Total contacts | Exposed vs. Unexposed | 6.03 (4.8-7.57) | 0.00 |
| Accident and emergency department (A&E) - Total contacts | (n) Pre-index Contacts | 1.73 (1.36-2.21) | 0.00 |
| Accident and emergency department (A&E) - Total contacts | (n) Pre-index Months in Community | 0.98 (0.97-0.99) | 0.00 |
| Accident and emergency department (A&E) - Total contacts | (n) Comorbidities | 1.81 (1.63-2.01) | 0.00 |
| Accident and emergency department (A&E) - MH | Exposed vs. Unexposed | 4.88 (3.78-6.29) | 0.00 |
| Accident and emergency department (A&E) - MH | (n) Pre-index Contacts | 1.89 (1.19-2.99) | 0.01 |
| Accident and emergency department (A&E) - MH | (n) Pre-index Months in Community | 0.98 (0.97-0.99) | 0.00 |
| Accident and emergency department (A&E) - MH | (n) Comorbidities | 1.8 (1.59-2.03) | 0.00 |
| Accident and emergency department (A&E) - SU | Exposed vs. Unexposed | 7.98 (5.71-11.17) | 0.00 |
| Accident and emergency department (A&E) - SU | (n) Pre-index Contacts | 2.26 (1.7-3.02) | 0.00 |
| Accident and emergency department (A&E) - SU | (n) Pre-index Months in Community | 0.97 (0.96-0.99) | 0.00 |
| Accident and emergency department (A&E) - SU | (n) Comorbidities | 2.04 (1.77-2.35) | 0.00 |
| Ambulance call-outs (SAS) - Total contacts | Exposed vs. Unexposed | 7.24 (5.96-8.8) | 0.00 |
| Ambulance call-outs (SAS) - Total contacts | (n) Pre-index Contacts | 1.69 (1.44-1.98) | 0.00 |
| Ambulance call-outs (SAS) - Total contacts | (n) Pre-index Months in Community | 0.97 (0.96-0.98) | 0.00 |
| Ambulance call-outs (SAS) - Total contacts | (n) Comorbidities | 1.85 (1.64-2.08) | 0.00 |
| Ambulance call-outs (SAS) - MH | Exposed vs. Unexposed | 7.75 (5.76-10.42) | 0.00 |
| Ambulance call-outs (SAS) - MH | (n) Pre-index Contacts | 4.46 (2.87-6.95) | 0.00 |
| Ambulance call-outs (SAS) - MH | (n) Pre-index Months in Community | 0.99 (0.98-1) | 0.05 |
| Ambulance call-outs (SAS) - MH | (n) Comorbidities | 2.03 (1.74-2.36) | 0.00 |
| Ambulance call-outs (SAS) - SU | Exposed vs. Unexposed | 7.58 (5.71-10.08) | 0.00 |
| Ambulance call-outs (SAS) - SU | (n) Pre-index Contacts | 2.92 (1.84-4.63) | 0.00 |
| Ambulance call-outs (SAS) - SU | (n) Pre-index Months in Community | 0.97 (0.96-0.98) | 0.00 |
| Ambulance call-outs (SAS) - SU | (n) Comorbidities | 2 (1.75-2.28) | 0.00 |
| Ambulance call-outs (SAS) - DD | Exposed vs. Unexposed | 8.28 (6.5-10.55) | 0.00 |
| Ambulance call-outs (SAS) - DD | (n) Pre-index Contacts | 2.33 (1.73-3.15) | 0.00 |
| Ambulance call-outs (SAS) - DD | (n) Pre-index Months in Community | 0.97 (0.96-0.98) | 0.00 |
| Ambulance call-outs (SAS) - DD | (n) Comorbidities | 1.91 (1.66-2.21) | 0.00 |
