# Supplementary material for: Health service contacts for mental health and substance use on release from prison: a retrospective population-based data linkage study
Source: BMJ Open. 2026 Feb 4;16(2):e107586. doi: 10.1136/bmjopen-2025-107586 (PMC12878195; doi:10.1136/bmjopen-2025-107586)
Supplement: Supplementary file 2 [file bmjopen-2025-107586.reviewer_comments.pdf]

PEER REVIEW HISTORY

BMJ Open publishes all reviews undertaken for accepted manuscripts. Reviewers are asked to complete a checklist review form and are provided with free text boxes to elaborate on their assessment. These free text comments are reproduced below.

ARTICLE DETAILS

Title (Provisional)

Post-imprisonment health service contacts for mental health and substance use: a retrospective population-based data linkage study

Authors

Connell, Catriona; Kjellgren, Richard; Savinc, Jan; Dougall, Nadine; Kurdi, Amanj; Watson, Jim; Haddow, Christine; Brown, Ashley; Parkes, Tessa; Hunt, Kate

---

VERSION 1 - REVIEW

**Reviewer**	**1**
**Name**	**Curtis, Michael**
**Affiliation**	**University of Calgary, Behaviours and Health Risks**
**Date**	**08-Sep-2025**
**COI**	**None**

Peer review for: Post-imprisonment health service contacts for mental health and substance use: a retrospective population-based data linkage study

Thanks for the opportunity to review this paper, this is as far as I recall my first time reading any study from the UK examining post-release health-service use among people leaving prison. Accordingly, this is an important paper and documents elevated rates of emergency, mental health and AOD-related health service contact among people leaving Scottish prisons, a pattern similar to many other countries around the world. This paper is generally quite well written and is likely to be of interest to some of BMJ Opens readers. I have the following minor suggestions for the authors which may improve the paper:

Background:

- The introduction, while noting AOD-related deaths, seems to focus more on mental health outcomes and service contacts. I wonder whether adding a little more on overdoses, especially opioids, may be warranted? Especially given this issue has been documented in Scotland: https://doi.org/10.1111/add.12969

- The second paragraph discusses contact with services within a range of timeframes in several countries; getting people into the service at least once following release from prison

is definitely an important point. Once there, keeping people engaged in care and treatment is the next challenge, which isn't really addressed. Perhaps there is scope to briefly discuss this, also? Of particular interest may be access and retention to OAT, given it's important role in reducing mortality (e.g., https://doi.org/10.1111/add.12536)

Methods:

- It is unclear where the Scottish Index of Multiple Deprivation (SIMD) variable comes from – is this something created by the external linkage agency?

- Similarly,

- Nice use of GitHub to provide access to what is a long list of codes across the different datasets. In the interest of helping a very keen reader follow and potentially replicate the work, it might be helpful to use the same delineations in the GitHub excel spreadsheet as in the paper – I assume that for the ICD-10 sheet, release_mh_bucket release_su_bucket release_mhsu_bucket and refer to MH, SU and DD, respectively?

- I presume the censor dates for exposed and unexposed participants was the date of death of the exposed participant.

- Great to see time in the community accounted for, and clearly and succinctly explained!

- It isn't explained anywhere how the Elixhauser confounder was derived. I assume this was created by the analysts/authors drawing on data from the different linked data sources?

- Can the authors provide any justification for the use of Poisson models instead of negative binomial models, given count data are often over dispersed? Perhaps means and variance data could be added to Table S3?

Results:

- I would consider bringing the data linkage flow diagram from the supplementary material into the main document.

- Several of the terms used in Table 2 and the descriptions of the results contained in Table 2 differ. Consistency of terms between the table and text would improve readability.

Discussion:

- "It is a public health priority that these individuals are identified and supported to engage with high-quality support that reduces later need for costly emergency services." It would be great to hear some ideas on how this could be improved in Scotland.

- "This suggests that among people released, SU needs may have a larger impact on acute and emergency health services than MH" – perhaps this is related to prompt return to substance use and the well-established period of elevated risk for opioid overdoses in the 90 days after release from prison?

- The paragraph at the bottom of page 15 suggesting prisons may have a facilitating effect on people contact healthcare post-release was a little surprising – while I cannot discount this possibility in Scotland given nothing has been published on it before, this would be surprising given the well documented challenges of continuity of care between prison and community in many developed countries. I find the sentence “This could be explained by escalating MH and SU problems during imprisonment and on release 25–27” to be a far more convincing explanation, especially in the context of well documented period of elevated risk for opioid overdose and challenges and stressors of community re-entry.

- The sentence “This suggests that either primary care and community service contacts do not result in adequate MH and SU care” may benefit from a little more nuance. Primary healthcare and community services are often over burdened, and people exiting prison often have several comorbidities, accordingly, general health may be ill-equipped to meet the many general health, SU and MH needs at once?

Reviewer 2

Name Bhavsar, Vishal

Affiliation Institute of Psychiatry Department of Health Service and Population Research, Department of Health Services and Population Research

Date 29-Nov-2025

COI None

Thank you. The analysis is well designed and clearly reported. I hope you find my suggestions helpful.

-could the addition/contribution be clarified, in terms of what is not known that this paper adds?

-relatedly, while the associations being estimated are clearly described, could a research question be included as I think this will help to clarify what the study is contributing.

-it might also be helpful to include a consideration of how the results of the might change policy or practice, given previous evidence is pretty clear that prisoners have greater needs, and use greater care despite barriers?

-Covariate adjustment implies that you are looking to account for confounding and assess the impact of incarceration on contacts for MH and SU. The limitations could eb expanded to consider whether your measured covariates capture all relevant causes of both incarceration and morbidity, and whether there may be uncaptured incarceration in your unexposed group which might have introduced bias.

VERSION 1 - AUTHOR RESPONSE

R	Comment	Response
1	The introduction, while noting AOD-related deaths, seems to focus more on mental health outcomes and service contacts. I wonder whether adding a little more on overdoses, especially opioids, may be warranted? Especially given this issue has been documented in Scotland: https://doi.org/10.1111/add.12969	In the manuscript introduction we give equal attention to SU and MH, with deaths, emergency care and onward service contacts for both discussed.<br><br>Reviewer 1 is correct that drug-related deaths is significant political and public health priority in Scotland. We have added two sentences contextualising the public health emergency in Scotland in relation to drugs, alcohol and suicide (pg. 3)
1	The second paragraph discusses contact with services within a range of timeframes in several countries; getting people into the service at least once following release from prison is definitely an important point. Once there, keeping people engaged in care and treatment is the next challenge, which isn't really addressed. Perhaps there is scope to briefly discuss this, also? Of particular interest may be access and retention to OAT, given it's important role in reducing mortality (e.g., https://doi.org/10.1111/add.12536)	We have added a paragraph highlighting to the evidence in support of medication assisted treatment, including from Scotland, and emphasised the importance of sustained engagement for full treatment benefits. We also highlight the need for the same for other substances and mental health treatment, acknowledging that the evidence for this is more limited. (pg. 3-4)
1	It is unclear where the Scottish Index of Multiple Deprivation (SIMD) variable comes from – is this something created by the external linkage agency?	We clarify what the SIMD is and that it was derived from the CHI database (pg. 6).
1	Nice use of GitHub to provide access to what is a long list of codes across the different datasets. In the interest of helping a very keen reader follow and potentially replicate the work, it might be helpful to use the same delineations in the GitHub excel spreadsheet as in the paper – I assume that for the ICD-10 sheet, release_mh_bucket release_su_bucket release_mhsu_bucket and refer to MH, SU and DD, respectively?	We provide further information on GitHub on interpreting the coding systems from the spreadsheet, and how they relate to our protocol, and the paper under review. Spreadsheet delineations relate to mental health and substance use-related codes; the dual diagnosis definition is based upon the co-existence of at least one MH and one SU code within the same contact record. Thus, the release_mhsu_bucket is a boolean variable which shows that the code is captured in either MH or SU buckets.
1	I presume the censor dates for exposed and unexposed participants was the date of death of the exposed participant.	We clarify the process of right censoring for exposed and unexposed individuals. We censored dates for exposed and unexposed individuals if the exposed individual died within the follow-up. We right censored unexposed individuals' follow-up time if they died prior to the end of follow-up or before their associated exposed individual (pg. 8).

1	It isn't explained anywhere how the Elixhauser confounder was derived. I assume this was created by the analysts/authors drawing on data from the different linked data sources?	We have provided a clarification regarding how the Elixhauser confounder was derived (pg. 8-9); the reviewer is correct in that two linked data sources were used to compile diagnoses from the pre-index period.
1	Can the authors provide any justification for the use of Poisson models instead of negative binomial models, given count data are often over dispersed? Perhaps means and variance data could be added to Table S3?	We have added a sentence to explain our reasoning (pg. 10 & Table S3). Poisson models with robust standard errors produce consistent estimates of the conditional mean service contact rate after adjustment, and the robust standard errors allow for valid inference in the presence of over-/under-dispersion - this allowed for a general model specification for all 28 models where the negative binomial variance structure assumption may or may not hold. We updated S3 to include the mean and variance of each outcome variable, and calculated the dispersion ratio according to Gelman and Hill (2007).
1	I would consider bringing the data linkage flow diagram from the supplementary material into the main document.	We have added the data linkage and participant flowchart to the main document (pg. 7 & Figure 1)
1	Several of the terms used in Table 2 and the descriptions of the results contained in Table 2 differ. Consistency of terms between the table and text would improve readability.	We have updated the terms used so that it's consistent between Table 2 and the text. (pg. 13-15)
1	"It is a public health priority that these individuals are identified and supported to engage with high-quality support that reduces later need for costly emergency services." It would be great to hear some ideas on how this could be improved in Scotland.	Implications for policy and practice section added.
1	"This suggests that among people released, SU needs may have a larger impact on acute and emergency health services than MH" – perhaps this is related to prompt return to substance use and the well-established period of elevated risk for opioid overdoses in the 90 days after release from prison?	This is a plausible explanation, however our follow up goes beyond the initial release period, and SU contacts were not only for opioids. We have added clarifying details to this paragraph to distinguish comparisons between exposed and unexposed groups, MH and substance use contact aIRRs, and MH and SU absolute rates of contact (pg. 17-18).
1	The paragraph at the bottom of page 15 suggesting prisons may have a facilitating effect on people contact healthcare post-release was a little surprising – while I cannot discount this possibility in Scotland given nothing has been published on it before, this would be surprising given the well documented challenges of continuity of care between prison and community in many developed countries. I find the sentence "This could be explained by	We have added a sentence indicating that our conclusion is that escalating MH and SU needs following prison/release may be the most plausible explanation, but that further research is needed to explain these patterns (pg. 18-19).

	escalating MH and SU problems during imprisonment and on release 25–27” to be a far more convincing explanation, especially in the context of well documented period of elevated risk for opioid overdose and challenges and stressors of community re-entry.	
1	The sentence “This suggests that either primary care and community service contacts do not result in adequate MH and SU care” may benefit from a little more nuance. Primary healthcare and community services are often over burdened, and people exiting prison often have several comorbidities, accordingly, general health may be ill-equipped to meet the many general health, SU and MH needs at once?	Clarified this sentence that it is likely a combination of service, context and population that make meeting the needs for this population challenging in primary as it is currently designed (pg. 19).
2	Could the addition/contribution be clarified, in terms of what is not known that this paper adds?	A clarifying statement has been added in the discussion (pg. 18) and conclusion (pg. 21) .
2	Relatedly, while the associations being estimated are clearly described, could a research question be included as I think this will help to clarify what the study is contributing.	We have added the research questions to assist with clarifying the study's contribution (pg 4)
2	it might also be helpful to include a consideration of how the results of the might change policy or practice, given previous evidence is pretty clear that prisoners have greater needs, and use greater care despite barriers?	We have added an ‘implications for policy, practice and research’ section.
2	Covariate adjustment implies that you are looking to account for confounding and assess the impact of incarceration on contacts for MH and SU. The limitations could be expanded to consider whether your measured covariates capture all relevant causes of both incarceration and morbidity, and whether there may be uncaptured incarceration in your unexposed group which might have introduced bias.	We added a sentence in discussing limitations acknowledging that there may be confounders that we were unable to adjust for (pg. 20). We clarify how we addressed minimal contamination of the unexposed group (pg. 9 and Fig 1), and restate this in results (pg. 11).

VERSION 2 - REVIEW

Reviewer 1

Name Curtis, Michael

Affiliation University of Calgary, Behaviours and Health Risks

Date 13-Dec-2025

COI

Thank you for the point by point responses to my previous comments. The revisions have addressed my previous concerns. Congratulations on this piece of work.

One final small items that can be addressed during copy editing, there appears to be a typo at the end of the sentence "Further, our results indicate a need to consider care provision beyond the immediate post-release period so that people have the opportunity to sustain engagement with high quality support for as long as necessary, on engage later post-release" on should be or?

Reviewer **2**

Name **Bhavsar, Vishal**

Affiliation **Institute of Psychiatry Department of Health Service and Population Research, Department of Health Services and Population Research**

Date **18-Dec-2025**

COI

Thank you for attending to my comments.
